# MicroRNA-29B (mir-29b) regulates the Warburg effect in ovarian cancer by targeting AKT2 and AKT3

**DOI:** 10.18632/oncotarget.5695

**Published:** 2015-10-20

**Authors:** Yue Teng, Yan Zhang, Kai Qu, Xinyuan Yang, Jing Fu, Wei Chen, Xu Li

**Affiliations:** ^1^ Department of Obstetrics and Gynecology, The First Affiliated Hospital of Xi'an Jiaotong University, Xi'an 710061, China; ^2^ Center for Translational Medicine, The First Affiliated Hospital of Xi'an Jiaotong University, Xi'an 710061, China; ^3^ Department of Hepatobiliary Surgery, The First Affiliated Hospital of Xi'an Jiaotong University, Xi'an 710061, China

**Keywords:** epithelial ovarian cancer, Warburg effect, microRNA, miR-29b, AKT

## Abstract

Epithelial ovarian cancer (EOC) is the most lethal and aggressive gynecological malignancy, and abnormal cellular metabolism significantly contributes to cancer onset and progression. Here, we report that miR-29b negatively regulates AKT2/AKT3 expression, causing HK2/PKM2 downregulation and leading to a decreased Warburg effect and slowed ovarian cancer progression. Compared to normal ovaries, ovaries with epithelial cancer exhibited lower miR-29b expression at both cellular/histological levels. Glucose consumption and lactate production experiments confirmed miR-29b's regulation of EOC metabolism. A luciferase reporter assay confirmed the direct binding of miR-29b to AKT2/AKT3 3′ UTRs. miR-29b silencing correlated with increased expression of AKT2/3, pAKT2/3, HK2, and PKM2. Pyruvic acid and NAD+/NADH levels also changed when miR-29b expression was suppressed; this effect could be blocked by specific AKT inhibitors, suggesting the miR-29b-AKT axis regulates the Warburg effect in ovarian cancer. In xenograft mouse models, miR-29b inhibited tumor formation *in vivo*. *In vivo* imaging also demonstrated that miR-29b agomir inhibited the relative uptake of ^18^F-FDG in the xenograft tumors, suggesting that miR-29b over-expression could negatively modulate tumor glucose metabolism *in vivo*. Taken together, our study suggests that miR-29b regulates the Warburg effect in EOC via AKT2/AKT3 and may provide novel options for future treatments for EOC.

## INTRODUCTION

Normal somatic cells use mitochondrial oxidative phosphorylation as their main metabolic pathway. Cancer cells, however, preferentially use a far less efficient pathway, aerobic glycolysis, to metabolize glucose [1, 2]. This phenomenon, termed the Warburg effect, is characterized by increased glycolysis and lactate production, even in the presence of oxygen [1]. Since Warburg's initial observation, the preference of cancer cells for glycolysis over energy-efficient oxidative phosphorylation has remained a mystery. Over half a century, several hypotheses have been proposed as possible explanations. However, the mechanism underlying this phenomenon remains largely obscure. Among the various cancer- and metabolism-related cellular signaling pathways, the PI3K/AKT pathway is a major signaling cascade that regulates glucose metabolism as well as controls cellular growth. AKT is an evolutionarily conserved serine/threonine kinase, and is also known as protein kinase B(PKB). Mammalian cells express three highly homologous AKT isoforms (AKT1, AKT2, and AKT3) that are encoded by separate genes; the amino acid sequences of these isoforms exhibit more than 80% identity [3]. AKT activation regulates glucose transporter gene expression and enhances hexokinase gene expression, thus promoting glucose capture. AKT also increases phosphofructokinase activity, and stimulates the glucose to lactate metabolic pathway, all of which converge to promote tumorigenesis [4, 5].

MicroRNAs (miRNAs) are a group of short, noncoding endogenous RNAs, approximately 22nt in length. The classic functioning of mature miRNAs involves imperfect complementary sequence pairing between an miRNA seed region and the 3′untranslated region (UTR) of the target gene, resulting in negative regulation of the target gene by either mRNA degradation or translational repression [6]. miRNAs play critical regulatory roles in various biological activities such as tumorigenesis and metabolism [7–10]. Unfortunately, little is known regarding the specific role of miRNAs in cancer metabolism, especially with respect to the Warburg effect.

Epithelial ovarian cancer (EOC) accounts for 25% of all the malignancies that affect the female genital tract and is the most lethal gynecological malignancy. Despite aggressive treatment, most EOC patients develop recurrent cancer, and cancer metastasis is one of the leading causes of death [11]. Thus, novel biomarkers for early diagnosis and effective therapeutic treatment will significantly improve the current treatment and prognosis of ovarian cancer.

Among the nearly 2,000 miRNAs identified in mammalian cells to date, miR-29b particularly aroused our interest. miR-29b, a member of the miRNA-29 family, has been shown to participate in both the onset and progression of various malignant tumors, including ovarian cancer [12–16]. miR-29b has been shown to be downregulated in ovarian cancer. miR-29b has also been reported to be involved in diverse physiological and pathological processes, including cell differentiation, cell cycle control, apoptosis, and cancer progression [17]. Furthermore, there is also evidenceofmiR-29b's role in cellular metabolism, including the regulation of both amino acid synthesis and insulin release [18, 19]. Using bioinformatics, we previously identified AKT2 and AKT3, both of which are key proteins in the AKT signaling pathway, as potential downstream target genes of miR-29b, indicating that miR-29b-mediated effects on the AKT signaling pathway is probably involved in cancer glycolysis and the Warburg effect. However, to date, no studies have investigated whether miR-29b plays a role in the Warburg effect of ovarian cancer. We believe that elucidating the specific roles of miR-29b in both the Warburg effect and the latent molecular mechanisms of ovarian cancer will benefit our theoretical understanding of human ovarian cancer and provide future clinical approaches to treating this disease.

## RESULTS

### miR-29b is differentially expressed in human ovarian cancers & normal ovaries and is involved in the regulation of ovarian cancer cell metabolism

**Figure 1:**
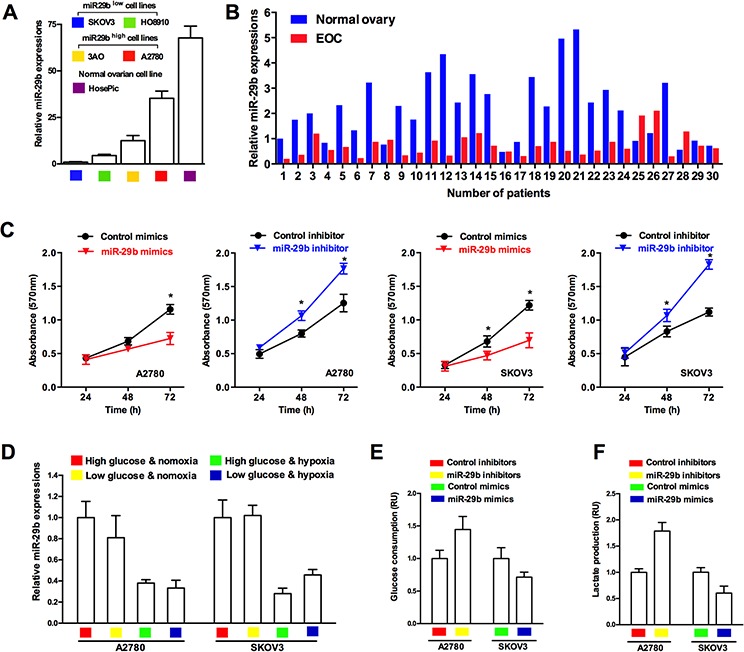
miR-29b is differentially expressed in human ovarian cancers & normal ovaries, and involved in regulating ovarian cancer cell metabolism **A.** Normal ovarian epithelial cells HOSEpiC shows highest endogenous miR-29b expression compared with 4 selected ovarian cancer cell lines; **B.** qPCR results shows that miR-29b in benign ovarian epithelia is much higher than those in human cancerous ovarian epithelia; **C.** MTT assay results reveal that miR-29b mimics transfection led to a decrease in absorbance (OD value) at 570 nm, while miR-29b inhibitors transfection led to an increase in absorbance (OD value) at 570 nm in both A2780 and SKOV3 cells; **D.** qPCR results indicate that miR-29b expression varies in different metabolic conditions in ovarian cancer cell lines A2780 and SKOV3. **E.** and **F.** Inhibition of miR-29b expression by target inhibitors transfection leads to increased glucose consumption and lactate production in A2780 cells, and over-expression of miR-29b by target mimics transfection causes decreased glucose consumption and lactate production in SKOV3 cells. Data are presented as means ± SEM, *n* = 3. *p < 0.05 versus control.

miR-29b was shown to be decreased in several malignant tumors, including ovarian cancer [16]. We investigated the expression of miR-29b in human ovarian cancer cells and human normal ovarian epithelial cells, to determine whether miR-29b is differentially expressed in cancerous and normal ovarian cells. As shown in Figure 1A, compared with the expression in four cancerous ovarian cell lines, miR-29b expression was significantly higher in the human normal ovarian epithelial cell line HOSEpiC. Furthermore, among the four human ovarian cancer cell lines, A2780 exhibited the highest endogenous miR-29b expression, and SKOV3, the lowest (Figure 1A). We then examined miR-29b and its latent downstream targets, AKT2 and AKT3 in 30 human ovarian cancer tissues and 30 matched normal ovarian tissues as negative controls. Similar to the results of the cellular experiments, the expression of miR-29b was much higher in the normal ovarian epithelia than in the human cancerous ovarian epithelia (Figure 1B). Moreover, MTT assay results showed that miR-29b also influenced ovarian cancer cell proliferation. Specifically, the over-expression of miR-29b via the transfection of target miRNA mimics led to a decrease in absorbance (OD value) at 570 nm in both A2780 and SKOV3 cells. In contrast, miR-29b knockdown via the transfection of target miRNA inhibitors led to an increase in the (OD value at 570 nm in the same cell lines (Figure 1C). A transwell migration assay and wound-healing assay also showed that miR-29b expression negatively correlated with ovarian cancer cell migration capacity, as demonstrated in Supplementary Figures S1 and S2. Because changes in cellular growth, proliferation, and migration are the basic outcomes of altered metabolism in cancer cells, we next explored whether miR-29b also regulated ovarian cancer cell metabolism. First, we tested the expression levels of miR-29b in different metabolic conditions (high/low glucose plus normoxia/hypoxia) in two representative ovarian cancer cell lines, A2780, with the highest endogenous miR-29b expression among the four cancer cell lines, and SKOV3, with the lowest endogenous miR-29b expression among the four cancer cell lines. As demonstrated in Figure 1D, miR-29b was differentially expressed under different metabolic conditions in both of the selected ovarian cancer cell lines, indicating the involvement of miR-29b in ovarian cancer metabolism. Next, to determine whether miR-29b is associated with the Warburg effect in ovarian cancer cells, we evaluated measures that reflect glucose metabolism, including glucose consumption and lactate production, in the A2780 and SKOV3 cell lines. Inhibition of miR-29b expression led to increased glucose consumption and lactate production in the A2780 cells, while over-expression of miR-29b decreased glucose consumption and lactate production in the SKOV3 cells (Figure 1E and 1F). These results confirm that miR-29b is involved in regulating the Warburg effect in ovarian cancer cells.

### miR-29b directly targets and thus negatively regulates AKT2 and AKT3

**Figure 2:**
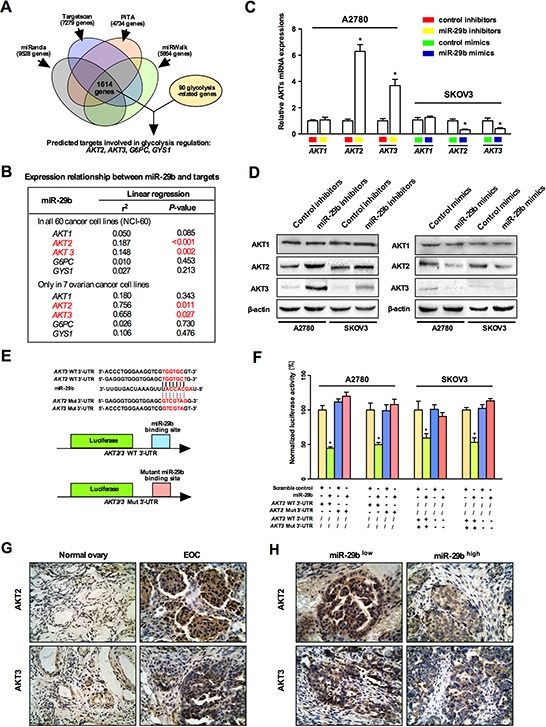
miR-29b directly targets and thus negatively regulates AKT2 and AKT3 **A.** A schematic shows the prediction and screening process of miR-29b downstream target gene involved in cancerous glycolysis regulation by a series of microRNA bioinformatics softwares; **B.** Expression analysis between miR-29b and predicted downstream target genes using the NCI-60 expression profiling data. Among the five selected genes, AKT2 and AKT3 showed a significantly negative correlation against miR-29b level; **C.** qPCR results indicate that miR-29b inhibition increased AKT2 and AKT3 levels in A2780 cells, and miR-29b overexpression decreased AKT2 and AKT3 levels in SKOV3 cells. While no change in AKT1 levels was observed in both conditions; **D.** Western blot results show that miR-29b inhibition increased AKT2 and AKT3 levels, and miR-29b overexpression decreased AKT2 and AKT3 levels in both A2780 and SKOV3 cells. While no change in AKT1 levels was observed in both conditions; **E.** Schematic representation of different AKT2/AKT3 3′UTR luciferase reporters used in the transfection experiments are depicted; **F.** SKOV3/A2780 cells were co-transfected with either miR-29b or control mimics and 200 ng pGL3 reporter construct containing wild type or miR-29b site mutated 3′UTR of AKT2/3. The relative firefly luciferase activities normalized with renilla luciferase were measured 48 hours post-transfection and results are plotted as percentage change over respective controls. **G.** Immunohistochemical results reveal that expression of AKT2 and AKT3 is higher in EOC epithelia than in normal ovarian epithelia; **H.** Immunohistochemical results indicate a negative correlation between miR-29b and AKT2/3 levels in EOC epithelia. All IHC images were photographed under 400× magnification. Data are presented as means ± SEM, *n* = 3. *p < 0.05 versus control.

Still, the concrete role of miR-29b in regulating the Warburg effect and the precise mechanism underlying this regulation remained unclear. To this end, we employed four miRNA target predicting websites (including miRanda, Targetscan, PITA, and miRWalk) to predict the downstream targets of miR-29b related to cancerous metabolism. As indicated in Figure 2A, a total of 1614 genes were identified by all four bioinformatics approaches; among these, 90 glycolysis-related genes were identified. Four of these genes, AKT2, AKT3, G6PC, and GYS1, were particularly interesting because their involvement in the regulation of glycolysis in cancer has been well documented. Next, we analyzed the relationships between miR-29b, these four putatively cancer glycolysis-regulating genes, and another key component of the AKT pathway, AKT1. As shown in Figure 2B, among these five genes, AKT2 and AKT3 were the most interesting, as they were significantly negatively correlatedwithmiR-29b levels not only in all 60 cancer cell lines but also in seven documented ovarian cancer cell lines (i.e., the seven ovarian cancer cell lines were selected according to their annotations and included IGROV1, OVCAR-3, OVCAR-4, OVCAR-5, OVCAR-8, SK-OV-3 and NCI_ADR_RES). Considering the findings described above, we then focused on AKT2 and AKT3, key proteins in the AKT signaling pathway, as potential downstream target genes of miR-29b. Thus, we hypothesized that miR-29b might play a role in the Warburg effect by directly targeting AKTs and negatively regulating their expression. To test our hypothesis, we employed miRNA mimics and inhibitors to specifically over-express and knock down endogenous expression of miR-29b in SKOV3 and A2780 cells, respectively. As shown in Figure 2C and 2D, the expression of AKT2 and AKT3 was significantly decreased after the cells were transfected with miR-29b mimics and was significantly increased at both the mRNA and protein levels after administration with miR-29b inhibitors. No change in AKT1 was observed at either the RNA or protein level, indicating that AKT1 is not involved in miR-29b's regulation of the Warburg effect in ovarian cancer cells. However, miR-29b negatively regulated both AKT2 and AKT3 expression in both of the selected ovarian cancer cell lines. Furthermore, we analyzed the 3′UTR sequences of AKT2/AKT3 as well as the mature chain sequence of miR-29b and found that the “seed region” of the miR-29b mature chain was fully complementary with and thus could potentially bind to the 3′ UTR sequences of AKT2 and AKT3 (Figure 2E). This observation raised the possibility that miR-29b might negatively regulate AKT2/AKT3 expression by directly binding to their 3′UTR sequences. A 3′UTR luciferase reporter assay confirmed that miR-29b directly bound to the 3′UTR of both AKT2 and AKT3. Briefly, ovarian cancer cells were transfected with miR-29b or control mimics in addition to a luciferase construct containing either the wild-type AKT2/AKT3 3′UTR or a mutant AKT2/AKT3 3′UTR (Figure 2E). Transfection of only the wild-type AKT2/AKT3 3′UTR significantly decreased (*P* < 0.05) luciferase expression. This suppressive effect of miR-29b was abolished by mutating the miR-29b site in the AKT2/AKT3 3′UTR (Figure 2F). Together, these results demonstrated that miR-29b binds directly to its complementary sequence motifs in the 3′ UTR of AKT2/AKT3, negatively regulating their expression. Moreover, immunohistochemistry (IHC) results showed that the expression of AKT2 and AKT3 was lower in normal ovarian epithelia than e in human cancerous ovarian epithelia (Figure 2G). Intriguingly, the ovarian cancer tissues that exhibited lower miR-29b expression also showed higher levels of AKT2 and AKT3 compared to their counterparts that exhibited higher miR-29b expression (Figure 2H). Also, a statistically significant negative correlation was found between miR-29b and AKT2 or AKT3 expression in EOC tissue (Supplementary Figure S3). These results indicated a negative correlation and a potential targeting relationship between miR-29b and AKT2/AKT3.

### AKT2 and AKT3 are positively correlated with ovarian cancer progression and glycolytic metabolism

**Figure 3:**
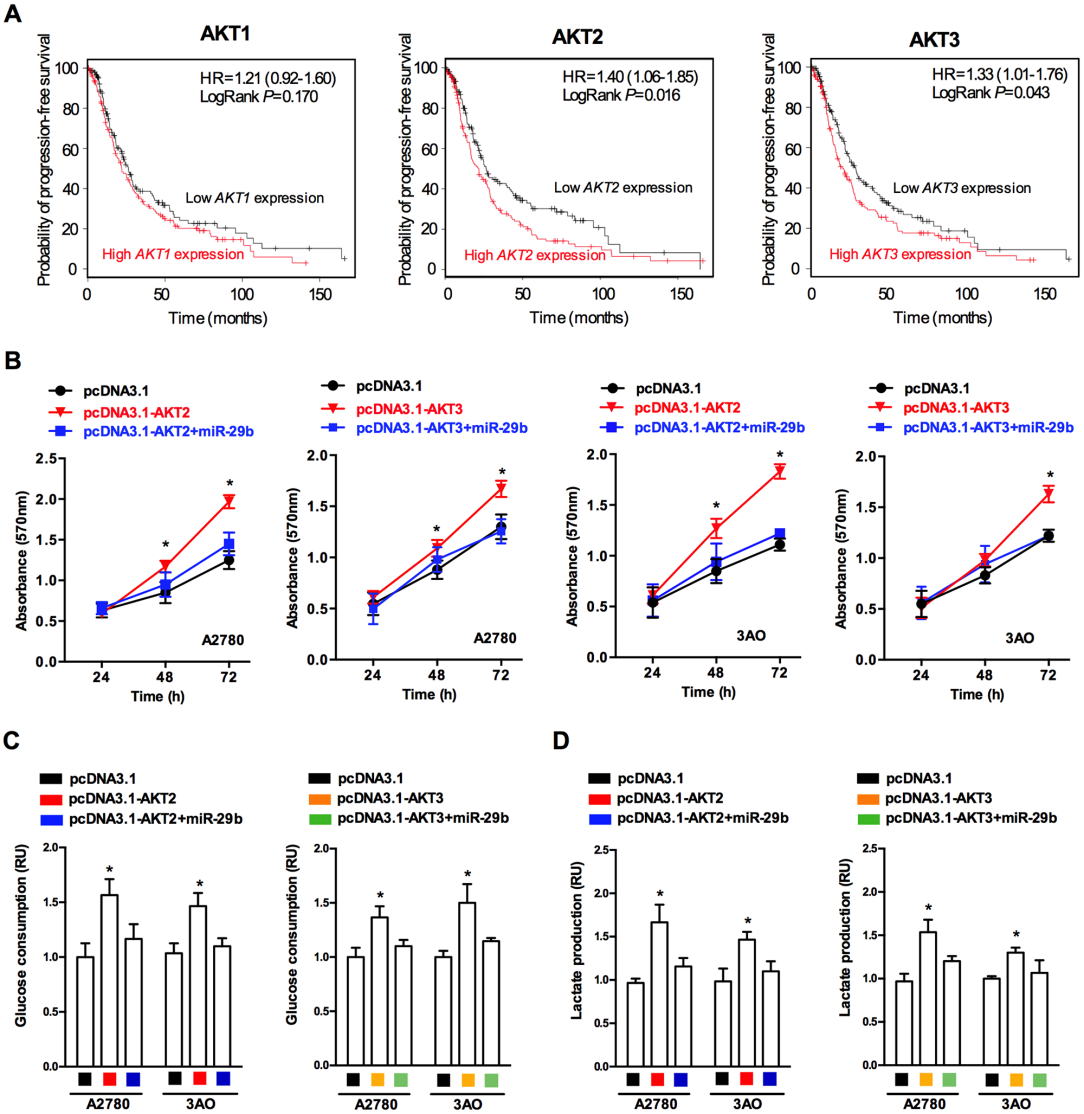
AKT2 and AKT3 are positively correlated with ovarian cancer progression and glycolytic metabolism **A.** Survival rate prediction results show that AKT2/3 expression negatively correlates with ovarian cancer patients' probability of progression-free survival, while no statistically significance was observed between AKT1 expression and probability of progression-free survival; **B.** MTT assay results reveal that both AKT2 and AKT3 overexpression led to an increase in absorbance (OD value) at 570 nm in both A2780 and SKOV3 cells, while simultaneous overexpression of AKT2/3 and miR-29b lead to no significant change in absorbance (OD value) at 570 nm in both A2780 and SKOV3 cells; **C.** AKT2/3 overexpression lead to increased glucose consumption in both A2780 and SKOV3 cells, while simultaneous overexpression of AKT2/3 and miR-29b lead to no significant change in glucose consumption in both A2780 and SKOV3 cells. **D.** AKT2/3 overexpression lead to increased lactate production in both A2780 and SKOV3 cells, while simultaneous overexpression of AKT2/3 and miR-29b lead to no significant change in lactate production in both A2780 and SKOV3 cells. Data are presented as means ± SEM. *p < 0.05 versus control.

AKT is activated by multiple mechanisms and is perhaps the most frequently activated onco-protein in human cancers. Among the three highly homologous AKT isoforms(AKT1, AKT2, and AKT3), most studies have focused on the role of AKT1 in cancer progression. To explore the effect of each AKT isoform in ovarian cancer, we analyzed the correlation between *in vivo* AKT1/AKT2/AKT3 levels and patients' probability of progression-free survival, based on data from a clinical study of patients suffering from ovarian cancer (http://kmplot.com/analysis/index.php?p=service&cancer=ovar). We selected patients with low cancer antigen 125 (CA-125) expression (average CA-125 below the lower quartile) for analysis; these patients were previously shown to have an increased risk of an abnormal metabolic state [20, 21]. As demonstrated in Figure 3A, AKT2/3 expression was significantly inversely correlated with the probability of progression-free survival, while a statistically significant relationship was not observed between AKT1 and the probability of progression-free survival, suggesting that AKT2 and AKT3 play a crucial role in the progression of ovarian cancer. Next, we determined the effects of AKT2 and AKT3 on *in vitro* ovarian cancer cell proliferation. The MTT assay results (Figure 3B) showed that over-expression of AKT2 and AKT3 enhanced cellular proliferation in the A2780 and SKOV3 ovarian cancer cell lines, while simultaneous over-expression of miR-29b and AKT2/3 led to no statistically significant change in cellular proliferation. Because altered proliferation is a basic outcome of altered metabolism in cancer cells, we then examined whether AKT2/3 expression affected the glycolytic metabolism of ovarian cancer cells. Important glucose metabolism events, including glucose consumption and lactate production, were examined in ovarian cancer cells over-expressing AKT2/3. As shown in Figure 3C and 3D, the over-expression of AKT2/3 increased glucose consumption and lactate production in both the A2780 and SKOV3 cells, while simultaneous over-expression of AKT2/3 and miR-29b caused no significant change in glucose consumption and lactate production in both the A2780 and SKOV3 cells, indicating that AKT2/3 overexpression leads to an increase in cancerous glycolytic metabolism, which could later be blocked by miR-29b overexpression. Collectively, these results indicate that AKT2 and AKT3 promoted ovarian cancer progression, probably by enhancing cancerous glycolytic metabolism.

### Activation of AKTs by miR-29b silencing contributes to the activation of key enzymes in the Warburg effect in ovarian cancer cells

**Figure 4:**
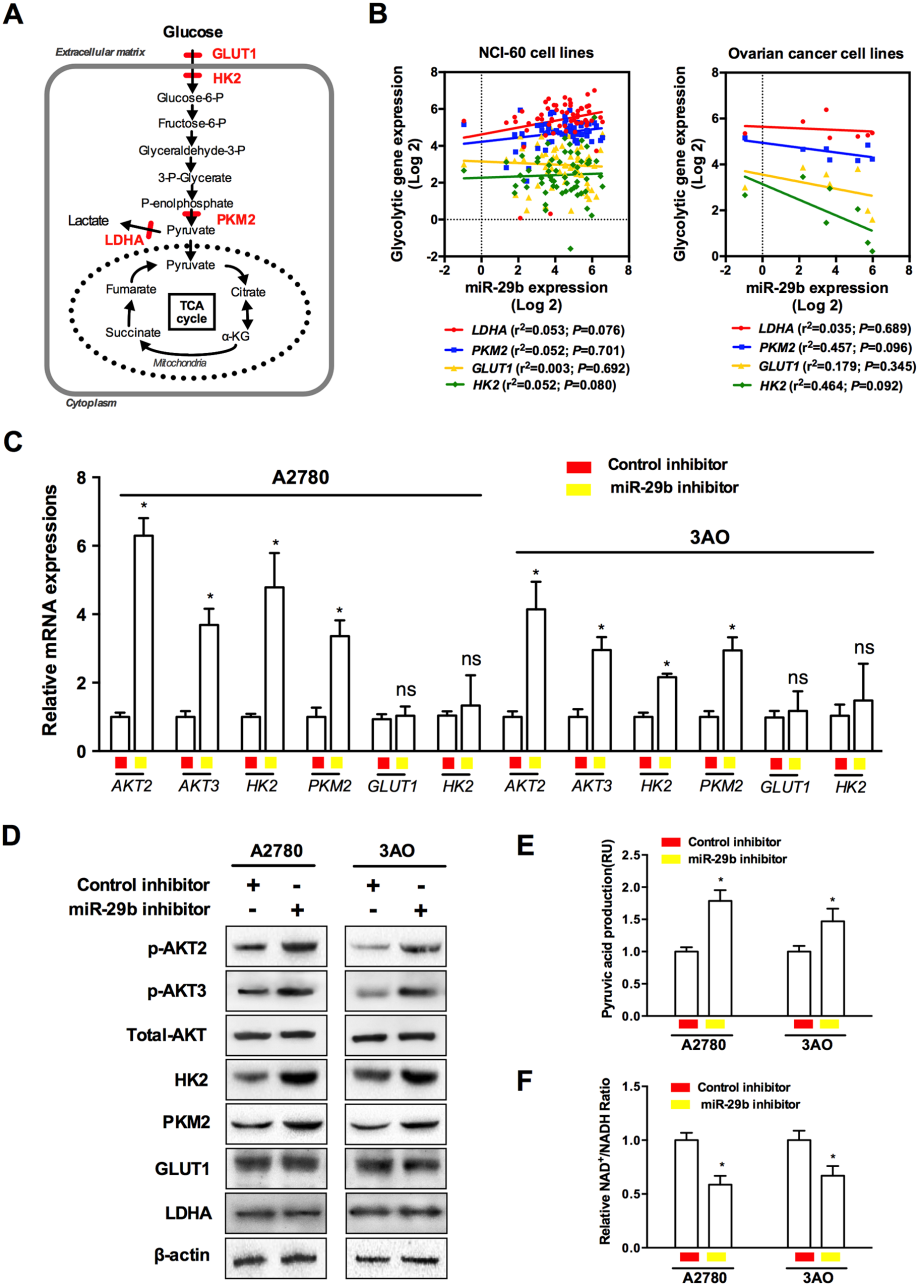
Activation of AKTs by miR-29b silencing contributes to the activation of key enzymes in the Warburg effect in ovarian cancer cells **A.** A schematic is provided to demonstrate various enzymes involved in regulating glycolytic metabolism of cancer cells; **B.** Correlation analysis between speed-limiting glycolytic genes (LDHA, PKM2, GLUT1, and HK2) expressions and miR-29b levels using the NCI-60 expression profiling data; **C.** and **D.** qPCR and WB results show changes in AKT2, AKT3 and four interested glycolytic enzymes when miR-29b is silenced by target inhibitors transfection in A2780 and 3AO cells at mRNA and protein levels, respectively; **E.** and **F.** Key glycolytic product pyruvic acid was increased by miR-29b silencing, while the ratio of NAD^+^/NADH decreased as ovarian cancer cells were transfected with miR-29b inhibitors in both A2780 and SKOV3 cells. Data are presented as means ± SEM. *p < 0.05 versus control.

By functioning at different levels, including the upregulation of membranous glucose uptake, post-translational modification of glycolytic enzymes, and the induction of the expression of certain glycolytic genes, the AKT pathway is a versatile regulator of cell growth, proliferation and metabolism as well as a potent stimulator of glycolysis [22]. The schematic in Figure 4A shows the various enzymes involved in the regulation of glycolytic metabolism in cancer cells. Among the various enzymes and isozymes that catalyze specific reactions in glycolysis, four crucial speed-limiting enzymes, GLUT1, HK2, PKM2, and LDHA favor aerobic glycolysis and growth in certain cancer types [23–26]. However, the molecular mechanisms of the miR-29b-AKT pathway in glycolytic metabolism in ovarian cancer cells remained unknown. Toward this end, we correlated the levels of these four glycolysis rate-limiting enzymes and miR-29b expression using the NCI-60 expression profiling data (GSE5846, http://www.ncbi.nlm.nih.gov/geo/query/acc.cgi?acc=GSE5846; and GSE26375, http://www.ncbi.nlm.nih.gov/geo/query/acc.cgi?acc=GSE26375). As demonstrated in Figure 4B, none of the four selected glycolytic genes was significantly correlated with miR-29b expression in the NCI-60 cancer cell lines or in the 7 ovarian cancer cell lines. Considering that relatively few samples were analyzed, which may influence statistical power to some extent, it is possible that statistically significant results could be obtained in future studies with a larger number of samples. However, in the 7 ovarian cancer cell lines subgroup, the results for two out of the four glycolytic enzymes, PKM2 and HK2,were closer to statistical significance than those for LDHA and GLUT1 (Figure 4B, *P*_PKM2_ = 0.096; *P*_HK2_ = 0.092). We then assessed the changes in the expression of AKT2, AKT3, and the four glycolytic enzymes caused by silencing endogenous miR-29b expression in the two ovarian cancer cell lines with constitutively high endogenous miR-29b expressions (A2780 and 3AO). As demonstrated in Figure 4C, the mRNA expression of AKT2/3, HK2, and PKM2 was upregulated when miR-29b was silenced, whereas no changes in GLUT1 and LDHA expression were observed. Similarly, p-AKT2/3, HK2, and PKM2 were all upregulated under the same conditions, whereas GLUT1 and LDHA were unaltered, as demonstrated by the western blotting results shown in Figure 4D. Moreover, the production of key metabolic products such as pyruvic acid and NAD^+^/NADH also changed as miR-29b expression was silenced. Pyruvic acid increased, whereas the ratio of NAD^+^/NADH decreased when the ovarian cancer cells were transfected with miR-29b inhibitors, as demonstrated in Figure 4E and 4F. These lines of evidence indicate that miR-29b silencing upregulated AKT2/3 levels and thus contributed to the activation of key enzymes in the Warburg effect. This could at least partially explain the latent mechanism of miR-29b's regulation of the Warburg effect in ovarian cancer cells.

### AKT inhibition blocks miR-29b silencing-induced AKT2/3 activation and glycolytic metabolic changes

**Figure 5:**
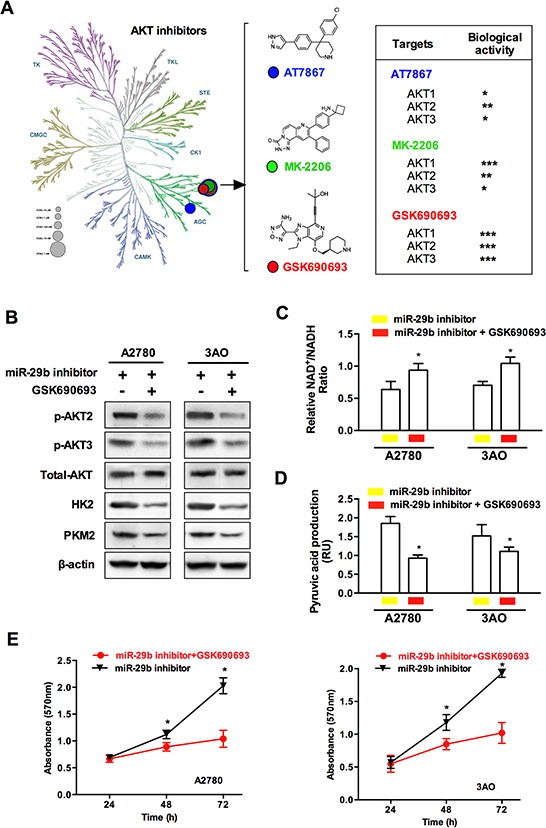
AKT inhibitor blocked miR-29b silencing-induced AKT2/3 activation and glycolytic metabolic change **A.** Here is a schematic showing various AKT inhibitors classified based on specific kinase targets in the serine/threonine kinase AKT molecule. Three pan-AKT inhibitors, including AT7867, MK-2206, and GSK690693 were selected and compared for their inhibiting bioactivities against each of the three AKT isoforms (AKT1, AKT2, and AKT3). **B.** WB results show that compared with those treated with miR-29b inhibitors transfection, an additional incubation of specific AKT inhibitor GSK690693 leads to a decrease in the protein levels of p-AKT2, p-AKT3, HK2, and PKM2 in both A2780 and 3AO cells. **C.** and **D.** the ratio of NAD^+^/NADH was increased, while key glycolytic product pyruvic acid decreased as specific AKT inhibitor GSK690693 was added in miR-29b-silenced A2780 and SKOV3 cells. **E.** An incubation of specific AKT inhibitor GSK690693 slowed cellular proliferation in miR-29b-silenced A2780 and SKOV3 cells. Data are presented as means ± SEM. *p < 0.05 versus control.

To confirm the involvement of the miR-29b-AKT axis in the regulation of the Warburg effect in ovarian cancer cells, we next silenced miR-29b and simultaneously employed AKT inhibitors to knock-down AKT expression; we then evaluated downstream changes in common glycolytic products. As demonstrated in Figure 5A, a variety of AKT inhibitors have been identified or artificially synthesized; these molecules target different parts of the serine/threonine kinase structure of the AKT molecule. Because an AKT inhibitor that specifically targets any of the three AKT isoforms (AKT1, AKT2, and AKT3) is not available, we analyzed the inhibitory effects of three commonly used non-specific AKT inhibitors, including AT7867, MK-2206, and GSK690693, on each AKT isoform (AKT1/AKT2/AKT3). As shown in Figure 5A, compared to the other two non-specific inhibitors, the pan-AKT inhibitor GSK690693 more strongly inhibited AKT2 and AKT3 and was thus selected for subsequent experiments. Western blotting showed that GSK690693 blocked the miR-29b silencing-induced activation of AKT2/3, HK2 and PKM2, confirming that the miR-29b-AKT axis regulates the activity of key glycolytic enzymes in ovarian cancer cells (Figure 5B). Moreover, compared to treatment with miR-29b inhibitors alone, the addition of GSK690693 also enhanced the NAD+/NADH ratio as well as decreased pyruvic acid production and cellular proliferation in both A2780 and 3AO cells, as shown in Figure 5C, 5D, and 5E. Together, these results indicate that GSK690693 blocked the miR-29b silencing-induced glycolytic metabolic changes and thus provide additional evidence of the function of the miR-29b-AKT axis in regulating the Warburg effect in ovarian cancer cells.

### Increased miR-29b expression suppresses xenograft tumor formation

**Figure 6:**
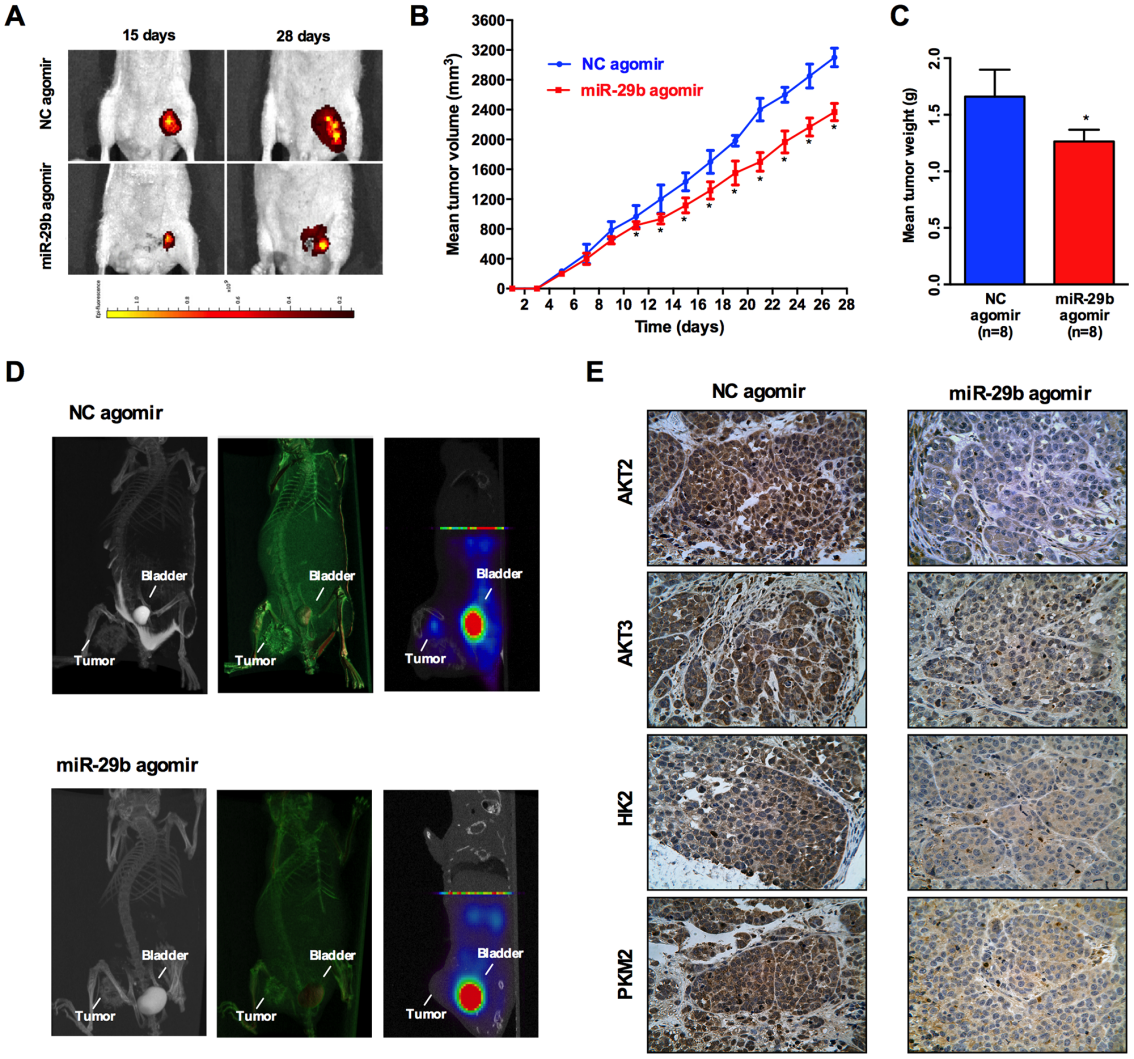
Increased miR-29b expression suppresses xenograft tumor formation An *in vivo* tumor model was established by subcutaneous injection of 1 × 10^6^ SKOV3 cells pre-transfected with Ago-miR-29b or Ago-miR-NC. **A.** Comparison of tumor growth (volume increase) between miR-29b group and the negative control group (control). The volume of solid tumors was monitored every other day and measured using vernier calipers. The tumor volume (V) was measured with a formula of V = (L × W^2^) × 0.5 (*n* = 8/group); **B.** and **C.** The tumors were weighed immediately after isolation from mice. The tumor weight was plotted between the two groups; **D.** Representative maximum intensity projection(MIP) and Complete volume rendering images from CT scanning, and ^18^F-FDG PET images of tumors(from left to right) in Ago-miR-29b and Ago-miR-NC treated mice. Sites of tumors and bladders are indicated in each picture. **E.** and **F.** Immunohistochemical analysis of AKT2, AKT3, PKM2, and HK2 in Ago-miR-29b or Ago-miR-NC treated mice xenograft tumors. All IHC images were photographed under 400× magnification.

To further evaluate the potential effect of miR-29b on ovarian cancer cell growth *in vivo*, SKOV3 cells with or without miR-29b over-expression were subcutaneously inoculated into the homolateral flanks of mice. The mean volume of the tumors of the miR-29b over-expressing group was significantly smaller than that of the tumors of the control group11 days after inoculation (Figure 6A). At the end of the experimental period, the mean wet weight of the tumors of the miR-29b over-expression group was significantly lower than that of the tumors of the control group (Figure 6B and 6C). Collectively, these data suggest that miR-29b may inhibit xenograft tumor formation of ovarian cancer cells *in vivo*. Moreover, because tumor cells exhibit significantly increased glucose uptake and elevated rates of glycolysis, uptake of the glucose analog ^18^F-FDG in tumors was compared with^18^F-FDG uptake in bladder tissue. Intriguingly, ^18^F-FDG uptake in xenograft tumors seemd to be much less vulnerable to treatment with miR-29b agomirs (Figure 6D). We also conducted IHC experiments of AKT2, AKT3, PKM2 and HK2 levels in xenograft tumor tissue sections. As indicated in Figure 6E, the expression of AKT2, AKT3, PKM2 and HK2 were significantly lower in the miR-29b agomir group compared with the NC agomir group, suggesting that miR-29b agomir treatment down-regulated *in vivo* AKT2/3 levels and further inhibited cancer cell glycolysis via downregulation of PKM2 and HK2 expression. Collectively, these data indicate that the miR-29b agomir is capable of inhibiting ^18^F-FDG uptake as well as glycolytic metabolism in xenograft tumors, suggesting that targeting miR-29b may prove to be an effective treatment to suppress ovarian cancer via the modulation of tumor glucose metabolism.

## DISCUSSION

Although miRNA signatures for ovarian cancer have been established, elucidation of the role of dysregulated miRNAs in ovarian carcinogenesis is in its infancy. miR-29b is a well-documented tumor-suppressing miRNA that is dysregulated in a number of human cancers, including prostate cancer, non-small-cell lung cancer, and breast cancer, among others [22, 25–28]. In this study, we showed that miR-29b reduced glycolysis in ovarian cancer cells and suppressed xenograft tumor formation in mouse models, revealing that miR-29b suppresses glucose metabolism in cancer cells. To our knowledge, this is the first report that this tumor-suppressing miRNA also plays a critical role in regulating cancer cell energy metabolism.

Activation of the AKT pathway is frequently observed in cancer. Using bioinformatics-based predictions, we found that miR-29b was involved in the regulation of AKT2 and AKT3 expression. Additional evidence for this regulation was provided by reporter gene assays and rescue experiments. We also identified HK2 and PKM2 as downstream targets of AKTs; HK2 and PKM2 catalyze two crucial steps of glucose metabolism, and both are key glycolytic enzymes for aerobic glycolysis. There is also evidence supporting the implications of AKT in the regulation of PKM2 as well as HK2 expression and their effect on proliferation and anti-apoptosis. For instance, Previous studies have demonstrated that hexokinases bound to the outer membrane of mitochondria produce an antiapoptotic activity [29, 30], which is strongly correlated with proliferation activity of malignant tumors [31]. The antiapoptotic effects of HK2 are known to be mediated by the PI3K downstream molecule AKT [30]. Akt signaling is reported to be important for the translocation of cytosolic HKII to the outer membrane of the mitochondria for antiapoptotic activity [32]. Also, both *in vitro* and *in vivo* experiments confirmed Akt2's role in regulating cell proliferation and PKM2 expression in a cell-autonomous manner in PTEN negative human HCC cell lines [33]. Similar to those findings, we demonstrated in our study that miR-29b suppresses glycolysis by targeting AKT-HK2/PKM2 in ovarian cancer cells. This finding, in addition to a previous report of miR-29b-mediatedcontrolof amino acid catabolism in human kidney cells, suggests a novel function of miR-29b in regulating cancer cell glucose metabolism [34].

Previous studies have suggested that miR-29b exerts its tumor-suppressing function by targeting oncogenes such as Bcl-2, Mcl-1, and MMP-2 [35–38]. Our findings here showed that the targeting of AKTs and their downstream effectors HK2 and PKM2 also contributes to miR-29b's anti-tumor function. Together, these results, suggest the possibility that in addition to regulating glucose metabolism, the miR-29b-AKT-HK2/PKM2 pathway also performs an important role in controlling tumourigenesis in ovarian cancer cells. These findings are in line with the notion that cancer cells employ aerobic glycolysis to generate biosynthetic materials that support cancer cell proliferation and build a tumor-favoring microenvironment that facilitates cancerous invasion and metastasis [39, 40]. Furthermore, they also add a novel role for miRNAs as a molecular link between carcinogenesis and tumor metabolism.

Additionally, the identification of miR-29b downregulation in ovarian cancer cells and tissues may increase the scope of potential therapeutics for ovarian cancer. Recent studies of the therapeutic uses of miRNAs have yielded encouraging results. The synthesis and use of miRNAs with extended *in vivo* half-lives and higher efficiency, such as the antisense oligonucleotides ‘antagomirs’, has generated favorable anti-tumor effects in previous studies of animal models [41]. Similarly, exogenous over-expression of miR-29b in ovarian cancer via the transfection of synthetic miR-29b oligonucleotides, infection with miR-29 viral vectors or other approaches to increase endogenous miR-29bmay be potent options for future clinical treatments of ovarian cancer. However, more detailed studies are needed to confirm this hypothesis.

## MATERIALS AND METHODS

### Cell lines and human tissue specimens

The human ovarian cancer cell lines SKOV3, A2780, HO8910(obtained from ATCC, Manassas, VA, USA), and 3AO (purchased from the Chinese Academy of Sciences Type Culture Collection, Shanghai, China) and the human ovarian epithelial cell line HOSEpiC (purchased from Shanghai Yaji Biotech, Shanghai, China) were maintained in high-glucose DMEM (Gibco, Invitrogen, Carlsbad, CA, USA) supplemented with 10% (v/v) fetal bovine serum at 37°C in a humidified 5% CO_2_ atmosphere. For the low-glucose treatment of the ovarian cancer cells, high-glucose DMEM and glucose-free DMEM were mixed at a volumetric ratio of 1:4 with supplementation with D-Mannitol (Sigma-Aldrich, St. Louis, Mo, USA)to maintain the same osmotic pressure of the cells cultured in the high-glucose condition. To induce hypoxia, cells were incubated at 1% O_2_, 5% CO_2_, and 94% N_2_ at 37°Cand 100% humidity in an HF100 hypoxia chamber (Heal Force, Hong Kong, China).

Human ovarian carcinomas and matched normal ovarian tissue samples were collected from patients at The First Affiliated Hospital, School of Medicine, Xi'an Jiaotong University, PR China. This study was approved by the Ethics Committee of The First Affiliated Hospital, School of Medicine, Xi'an Jiaotong University, China. Written consent was obtained from the study participants.

### Bioinformatics analysis

The targets and binding sites of miR-29b were predicted using several online programs that differed with respect to their databases and algorithms, including miRBase (http://www.mirbase.org/index.shtml), miRanda (http://www.microrna.org/microrna/home.do), miRWalk (http://www.umm.uni-heidelberg.de/apps/zmf/mirwalk/index.html), and TargetScan (http://www.targetscan.org/); AKT2 and AKT3 were predicted to be downstream target genes of miR-29b by all of these programs, and this was further validated by experiments.

### microRNA and transient transfection

The miR-29b mimics, control mimics, miR-29b inhibitors, and control inhibitors were all purchased from RiboBio (Guangzhou, China). Ovarian cancer cells were seeded into 6-well plates until 50%–60% confluent and transiently transfected with 60 nM control or miR-29b mimics or with 120 nM control or miR-29b inhibitors using X-treme GENE siRNA Transfection Reagent (Roche, Indianapolis, IN, USA) according to the manufacturer's instructions. After 48 h of miRNA transfection, the cells were harvested for further studies.

### Ago-miR and transfection

Stabilized miRNAs (miR-29b agomir and NC agomir) were purchased from RiboBio (Guangzhou, China). SKOV3 cells were transfected with 100 nM of miR-29b agomir or NC agomir (stabilized miRNA) by nucleofection with program T-028 of Nucleofector II (Amaxa Biosystems, Gaithersburg, MD, USA). After the transfection, the cells were incubated in high-glucose DMEM for 24 h at 37°C for recovery. Then, the cells were collected and washed with ice-cold 1 × PBS three times and suspended in 1 × PBS at a concentration of 1 × 10^7^ cells/ml for subsequent animal injections.

### Cell proliferation

Cell proliferation was assessed using the 3-[4,5-dimethylthiazol-2-yl]-2,5-diphenyltetrazolium bromide (MTT) (Amresco, Solon, OH, USA) assay. 5,000 cells/well were seeded in 96-well plates in high-glucose DMEM and routinely cultured for 72 h. The cells were then incubated in 20 μl MTT solution at a concentration of 5 mg/ml for 4 h at 37°C and lysed in 150 μl of dimethyl sulfoxide (DMSO) for 10 min at room temperature. A microplate reader measured the absorbance in each well at 570 nm.

### Detection of glucose consumption and lactate production

Glucose consumption and lactate production were analyzed as described previously [42]. Glucose levels were determined using a glucose assay kit (Sigma-Aldrich, St. Louis, Mo, USA). Lactate levels were determined using a lactate Assay kit (Biovision, Mountain View, CA).

### Measurement of intracellular NADH/NADPH by HPLC

The NAD^+^/NADH (μM) ratio was determined using an NAD^+^/NADH assay kit(Cat No: KGT6100-1, Keygenbio, Nanjing, China)according to the manufacturer's instructions. In brief, 200 μl 1 × PBS and 200 μl buffer (pH = 8.0) were added to standard NADH to createa1 mM NADH standard stock solution, which was later diluted to prepare a series of standard dilutions at concentrations of 1 μM, 0.3 μM, 0.1 μM, 0.03 μM, and 0.01 μM. The UV absorbance at 570 nm of each dilution was detected via HPLC to obtain the standard absorbance curve. Ovarian cancer cells were cultured in 96-well plates at a density of 5 × 10^5^ cells/well. After the cells in each well were washed with 1 × PBS and buffer (pH = 8.0),they were incubated with 200 μl NAD^+^ extraction solution/NADH extraction solution at room temperature for 15 min to extract intracellular NAD^+^/NADH. Then, 200 μl of NADH extraction solution/NAD^+^ extraction solution was added into each well to neutralize the sample pH. After the reaction, all of the solution was withdrawn from each well and 150 μl of DMSO was added. Then, the UV absorbance at 570 nm was detected for each sample via HPLC.

### Pyruvic acid production assay

Pyruvic acid is a key product of anaerobic cellular metabolism. Intracellular pyruvic acid production was detected with a pyruvic acid kit (A081, JianCheng Bio, Nanjing, China) according to the manufacturer's instructions. Briefly, ovarian cancer cells were cultured in 96-well plates at a density of 5 × 10^5^ cells/well. The UV absorbance at 505 nm was detected via HPLC for each sample. Pyruvic acid production was calculated with the following formula: Intracellular pyruvic acid production = (Sample OD–Blank OD)/(Standard OD–Blank OD)× Standard Concentration.

### Animals and xenograft transplantation

All experimental procedures involving animals were approved by the Ethics Committee of The First Affiliated Hospital, School of Medicine, Xi'an Jiaotong University, China. BALB/c mice (female and 4weeksold) were housed in SPF barrier facilities under a 12 h light/dark cycle. The xenograft growth model was created as previously described [43–45]. BALB/c female mice (2 groups, each group with at least eight mice, *n* = 8) were inoculated with SKOV3 cells via the subcutaneous injection of 1 × 10^6^ cells (100 μl) into the left flank. Xenograft growth was measured every 2 days beginning at day 5. Xenograft volume (V) was monitored by measuring the length (L)and width (W)with calipers and was calculated as V = (L × W^2^)/2. The tumors were isolated from mice with a surgical scissor at day 27, weighed, and then preserved in 4% PFA at 4°C for future IHC analysis.

### ^18^F-FDG small-animal PET-CT imaging

^18^F-FDG was generously provided by the PET-CT Center of the First Affiliated Hospital of Xi'an Jiaotong University, China. The ^18^F-FDG small-animal PET-CT imaging procedure was performed as previously described [46]. We used a prototype small-animal PET/CT system to acquire the PET-CT data. In the PET/CT system, a micro focus X-ray tube (L9181-02, Hamamatsu, Japan) and a high resolution flat panel X-ray detector (Dexela1512, Dexela, UK) were used in the CT sub-system, and the PET sub-system employed four basic detector modules (BDMs) developed by the Huazhong University of Science and Technology [47]. The area of each BDM was 53 mm × 53 mm, and two BDMs comprised a large detector with the area of 106 mm × 53 mm. Two such large detectors were placed facing each other with a distance of 50 mm, forming a dual-head PET system for mice imaging. 25 μL 18F-FDG (100 mCi) and 50 μL iohexol (15 mg) in 200 μL of saline were injected into the tail vain of each mouse. PET/CT data were acquired using our small-animal PET/CT system when the mice were fully anesthetized by isoflurane. PET data were acquired for 20 min for each mouse under continuous anesthesia 60 min after the 18F-FDG injection, and then the CT scanning were performed to acquire the anatomical images. The OSEM method was used for PET reconstruction with 5 subsets and 6 iterations, and the FDK method GPU acceleration was employed for CT reconstruction [48]. After reconstruction, the image analysis and fusion were performed using the 3DMed (http://www.mitk.net/; available on 2015-09-01) and Amide (http://amide.sourceforge.net/; available on 2015-09-01) softwares.

### Quantitative real-time PCR

Total RNA was isolated using TRIzol reagent (Invitrogen, Carlsbad, CA, USA) according to the manufacturer's instructions. To detect mRNA, first-strand cDNA was synthesized using a PrimeScript RT reagent kit (Perfect Real Time; Takara, Dalian, China). Quantitative real-time PCR was then carried out using SYBR Premix Ex Taq™ II (Takara) on a CFX96 real-time PCR System (Bio-Rad, Hercules, CA, USA). β-actin was used as an internal control to normalize the experimental results. The primers used are listed in Table 1. miR-29b levels were detected using a TaqMan microRNA kit (Applied Biosystems) and normalized to small nuclear RNA (Rnu6), which served as a control. The data were expressed as Log 2-fold changes relative to miRs/U6 snRNA levels. The primers for miR-29b and U6 reverse transcription and amplification were designed by and purchased from RiboBio Co., Ltd. (Guangzhou, China).

**Table 1:** Primers list

Genes	Primer Sequence (5′-3′)	Experimental use
AKT2	F: GAGGTCATGGAGCACAGGTT R: CTGGTCCAGCTCCAGTAAGC	Real-time PCR
AKT3	F: GGCGAGCTGTTTTTCCATTTG R: GGCCATCTTTGTCCAGCATTAG	Real-time PCR
HK2	F: TGTGCGTAATGGCAAGCGGAGG R: CCACGGCAACCACATCCAGGTC	Real-time PCR
PKM2	F: CCACTTGCAATTATTTGAGGAA R: GTGAGCAGACCTGCCAGACT	Real-time PCR
beta-actin	F:TCCCTGGAGAAGAGCTACGA R:AGCACTGTGTTGGCGTACAG	Real-time PCR
miR-29b	Mature chain: UAGCACCAUUUGAAAUCAGUGUU RT primer: CTCAACTGGTGTCGTGGAGTCGGCAATTCAGTTGAGAACACTGA F: ACACTCCAGCTGGGTAGCACCATTTGAAATC R: TGGTGTCGTGGAGTCG	Real-time PCR
U6	RT primer: AACGCTTCACGAATTTGCGT F: CTCGCTTCGGCAGCACA R: TGGTGTCGTGGAGTCG	Real-time PCR

### Western blot analysis

Cell lysates were collected using mammalian protein extraction reagent (Pierce, Rockford, IL, USA) with protease inhibitors (Roche, Indianapolis, IN, USA). The protein concentration of each sample was determined using a BCA-200 protein assay kit (Pierce, Rockford, IL, USA). The proteins were resolved on 10% SDS-polyacrylamide gels and transferred onto nitrocellulose membranes. The membranes were blocked in blocking buffer (5% non-fat milk in TBST). Rabbit anti-human AKT2 (ab39663, Abcam, Cambridge, UK), AKT3(ab152157, Abcam, Cambridge, UK), p-AKT2 (phospho S474, ab97727, Abcam, Cambridge, UK), p-AKT3 (phospho S472, A4716, Biorbyt, Cambridge, UK), and mouse anti-human β-actin (#3700S, CST, MA, USA) were used and were incubated with the membranes overnight at 4°C at dilutions of 1:800, 1:800, 1:600, 1:600 and 1:2000, respectively. After the blots were washed with TBST, they were visualized with anti-rabbit or anti-mouse IgG conjugated with peroxidase (HRP) and ECL reagents (Pierce, Rockford, IL, USA).

### Luciferase reporter assay

The *AKT2* and *AKT3* 3′UTRs containing the predicted *miR-29b* target sequence were amplified from genomic DNA (A2780 and SKOV3 cells) and cloned into the pGL3 firefly luciferase control vector (Promega, Madison, WI) at the XhoI restriction site immediately downstream of the luciferase reporter gene. To generate *AKT2/AKT3* 3′UTRs with a mutant target sequence, transversion mutations of 7 nucleotides were made at the *miR-29b* seed region complementary sites as shown in Figure 3C. Post-transcriptional inhibition of the luciferase reporter gene by *miR-29b* was assayed in A2780 and SKOV3 cells. Briefly, ovarian cancer cells were seeded into 24-well plates and cultured until 70%–80% confluent. The cells were then co-transfected with either miR-29b or control mimics at a 120 nM final concentration or with 200 ng of pGL3 reporter construct containing wild-type or miR-29b with the mutated AKT2/AKT3 3′UTR using the X-treme GENE HP DNA Transfection Reagent according to the manufacturer's recommendations (Roche, Indianapolis, IN, USA). The transfections were performed in quadruplicate. Relative firefly luciferase activity, which was normalized with Renilla luciferase, was measured 48 h post-transfection using a dual-luciferase reporter gene assay system (Promega, Madison, WI, USA), and the results were plotted as percentage change over the respective control.

### Immunohistochemistry assays

IHC assays were performed on single serial sections prepared from surgical samples. The slides were probed with a primary antibody against AKT2 (1:50;ab39663, Abcam, Cambridge, UK), AKT3 (1:200; ab152157, Abcam, Cambridge, UK), HK2 (1:200; #AP8140a, Abgent, San Diego, US), or PKM2 (1:200; ab38237, Abcam, Cambridge, UK) and then incubated with HRP-conjugated IgG (1:500; Invitrogen, Carlsbad, CA, USA). The proteins were visualized *in situ* with 3,3′-diaminobenzidine. Moderate- and high-power fields (200× and 400×) were photographed using an Eclipse 80i (Nikon, Tokyo, Japan) microscope.

### Statistical analysis

All of the experiments were performed at least in triplicate. Each experiment was independently performed at least 3 times. The data were presented as the mean ± standard deviation (SD) and analyzed using SPSS 19.0 and GraphPad Prism 5 software. Statistical significance was assessed using two-tailed unpaired Student's *t*-test. Differences were considered statistically significant when a *P* value was less than 0.05.

## SUPPLEMENTARY FIGURES


