# Supplementary material for: MicroRNA-29B (mir-29b) regulates the Warburg effect in ovarian cancer by targeting AKT2 and AKT3
Source: Oncotarget. 2015 Oct 20;6(38):40799–814. doi: 10.18632/oncotarget.5695 (PMC4747369; doi:10.18632/oncotarget.5695)
Supplement: Supplementary file 1 [file oncotarget-06-40799-s001.pdf]

## SUPPLEMENTARY FIGURES

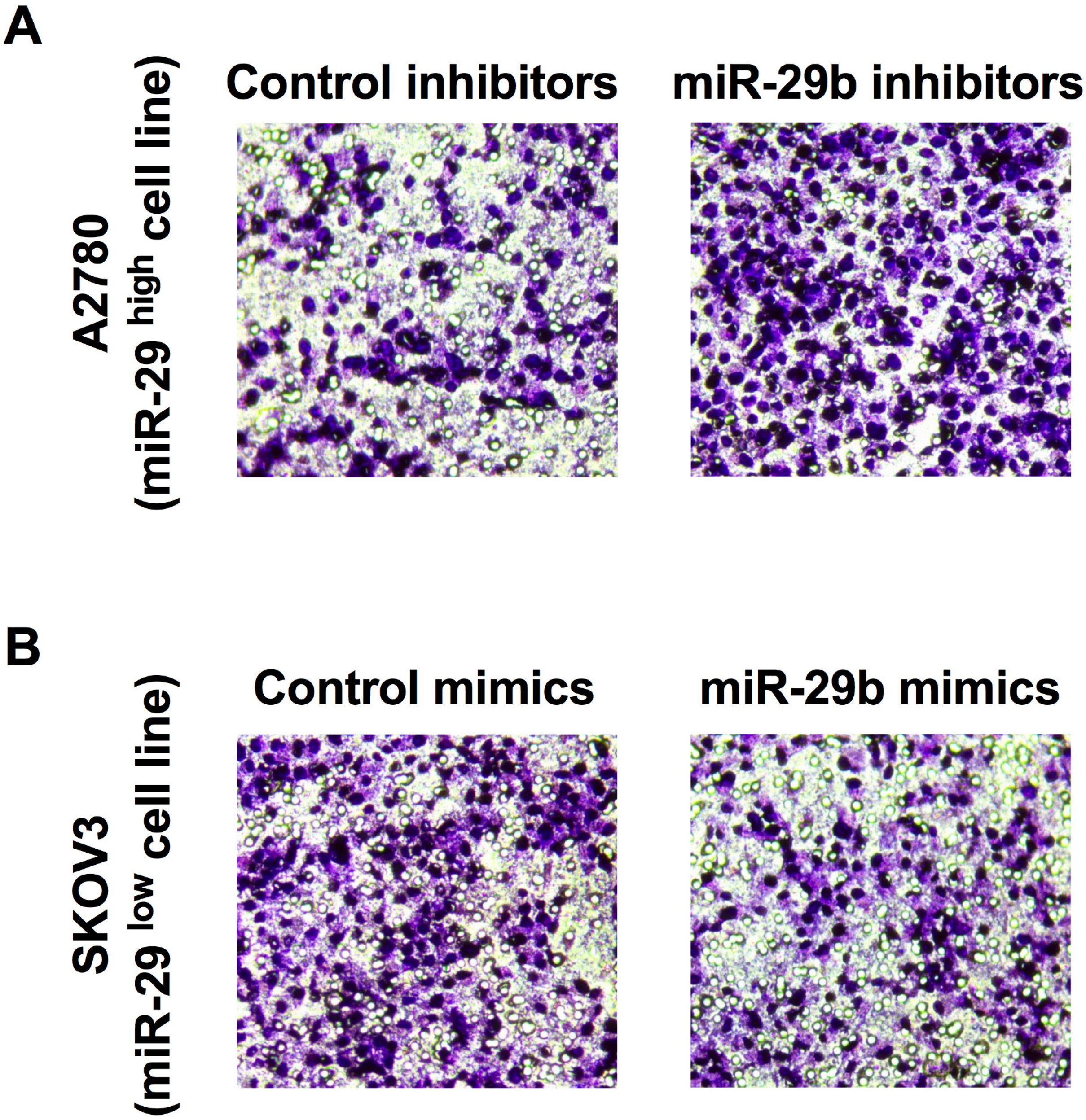


**Supplementary Figure S1: miR-29b expression negatively correlates with ovarian cancer cell migration capacity (Transwell migration assay). A.** In a transwell migration assay, target inhibitors-mediated miR-29b knockdown in A2780 cells leads to a significant increase in migrating capacity. **B.** In a transwell migration assay, target mimics-mediated miR-29b overexpression in SKOV3 cells leads to a significant decrease in migrating capacity. The images of the transwell migration assays were photographed under 400× magnification.

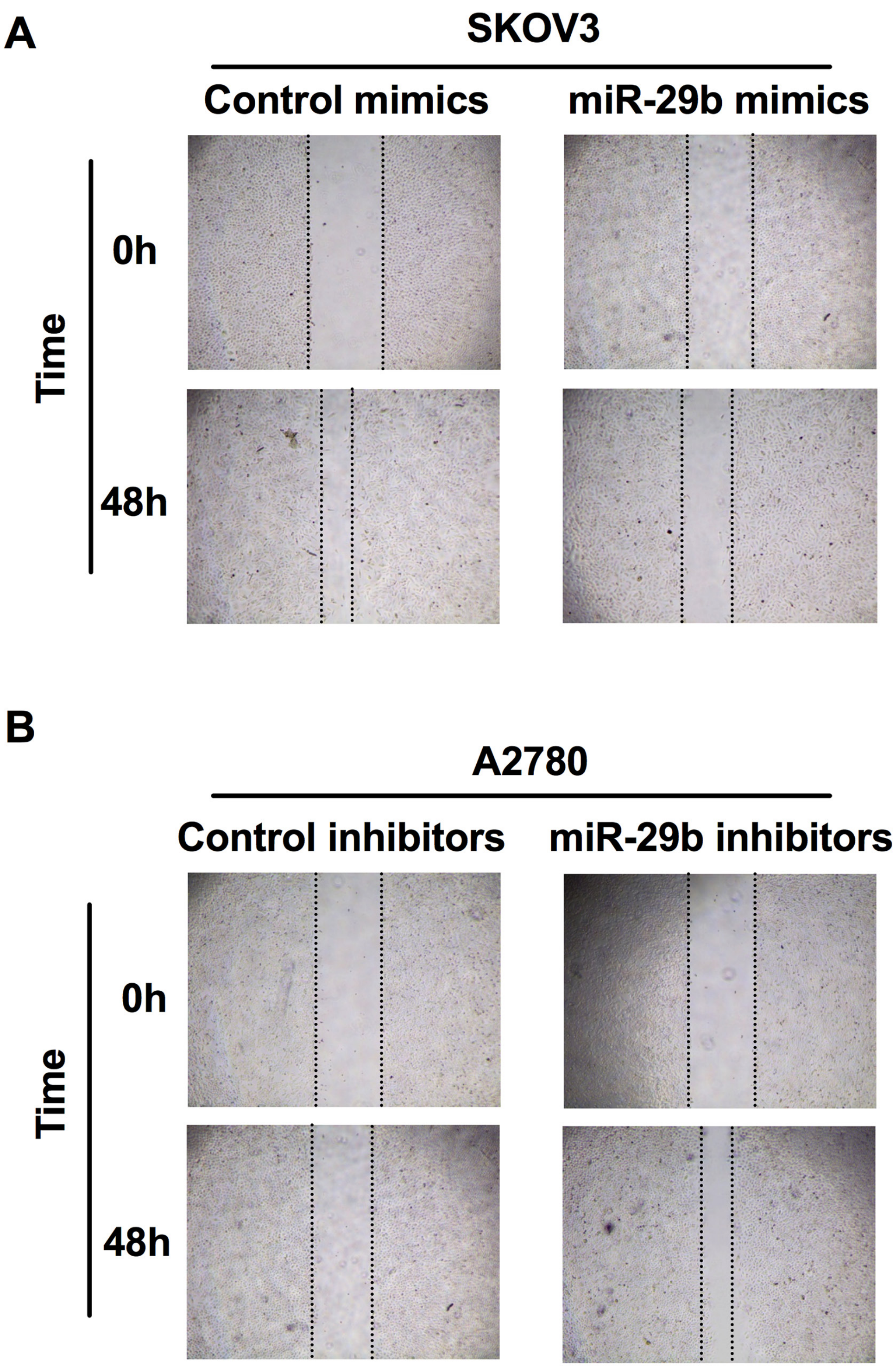


**Supplementary Figure S2: miR-29b expression negatively correlates with ovarian cancer cell migration capacity (Wound healing assay). A.** Representative images of wound healing show that mimics-mediated miR-29b overexpression in SKOV3 cells slows the wound healing. **B.** Representative images of wound healing show that inhibitors-mediated miR-29b knockdown in A2780 cells increases wound healing rate. The images of the wound healing assays were photographed under 100× magnification.

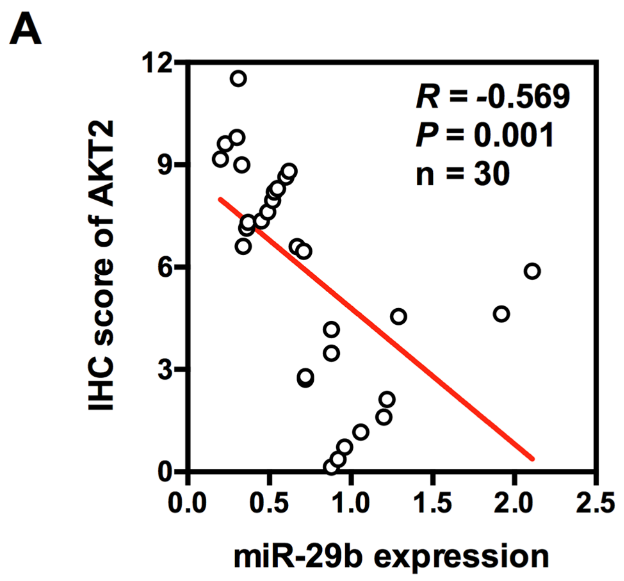


**Supplementary Figure S3: Association between expression of miR-29b and AKTs in ovarian cancer tissues. A.** Association between expression of miR-29b and AKT2 in 30 ovarian cancer samples(correlation coefficent$R$ = −0.569, $P$ = 0.001). **B.** Association between expression of miR-29b and AKT3 (correlation coefficent $R$ = −0.550, $P$ = 0.002).
